# Manipulation of particle microorganism association for improved UV disinfection of surface waters

**DOI:** 10.1038/s41598-025-01101-3

**Published:** 2025-05-22

**Authors:** Mehrnaz Soleimanpour Makuei, Nicolas Peleato

**Affiliations:** Department of Civil Engineering, University of British Columbia, Okanagan, Canada

**Keywords:** Particle-microorganism association, Ultraviolet disinfection, Zeta potential manipulation, Biodosimetry, Pre-treatment, Environmental impact, Civil engineering

## Abstract

The performance of ultraviolet (UV) disinfection is subject to the quality of water supplied to the reactor, which could often be poor in facilities lacking adequate pre-treatment. Particulate matter in low-quality water can interfere UV disinfection by shielding microorganisms from light through particle-microorganism (p-m) associations. This study investigates Zeta Potential (ZP) manipulation as a pre-treatment to improve UV effectiveness by reducing p-m associations. ZP manipulation is hypothesized to free microorganisms from particulate attachments, increasing their UV vulnerability. Water samples from a drinking water treatment plant applying UV disinfection without pre-filtration were altered for ZP, achieving five different ZP levels. A protocol was developed to distinguish between viable microorganisms attached to particles and free-floating microorganisms. UV experiments were conducted to establish the relationship between UV efficiency and ZP. Results indicated that neutral ZP results in the highest p-m association and lowest disinfection achieved. Disinfection kinetic studies revealed that highly negative ZP enhanced UV efficiency as delivered UV dose increased due to dominant repulsive forces. This study demonstrated that optimizing ZP effectively controlled the degree of p-m association for both viruses and bacteria, which could be a viable approach for mitigating p-m association and leveraged for advancements in UV disinfection.

## Introduction

Ultraviolet (UV) disinfection occurs when light in the germicidal wavelength range is radiated with sufficient intensity and time to disinfect microorganisms^1,2^. Unlike water disinfection strategies involving oxidizing agents, UV offers unique advantages, including lower potential for environmental impacts, high efficacy for oxidation-resistant microorganisms, such as protozoa, and avoiding the production of disinfection by-products and infrastructure-related issues such as handling and space constraints^3,4^. However, the performance of UV disinfection is more challenging to monitor since no measurable residual disinfectant is present. Compounding uncertainty in disinfection achieved is the significant influence of dynamic characteristics of the UV lamp, radiation pattern, reactor design, and reactor hydraulics on disinfection performance. Furthermore, the performance of UV systems is subject to the quality of water supplied to the reactor. Notably, suspended and dissolved substances can interfere with the delivery of UV light to organisms and lead to lower system performance if not adequately accounted for in design and operation^1,5,6^.

It is generally recommended that UV be applied after pre-treatment (e.g. filtration) to reduce the impact of particulate matter^7^. Many water treatment plants, especially small-scale systems, often lack comprehensive pre-treatment processes due to financial constraints, lack of required maintenance, and lack of operator capacities^8,9^, which increases the likelihood of higher levels of dissolved and suspended matter in the reactor. Additionally, extreme weather conditions driven by climate change, such as heavy precipitation, drought, and wildfires, can compromise the consistency of water quality in any given system^10,11^. The rapid development and decreasing costs of UV light-emitting diodes (UV-LED) are likely to increase the use of UV disinfection in small systems and Point Of Use (POU) applications due to cost-effectiveness and lower input energy requirements^12^. As such, UV disinfection is expected to be utilized increasingly in scenarios where complete or robust pre-filtration is not practiced, or it is highly desirable to avoid this requirement.

One consequence of increasing particle concentrations in UV reactor influent is the potential for particle-microorganism (p-m) association. These associations, arising through various mechanisms, such as adsorption, scattering or enmeshment^13,14^, hinder the exposure of microorganisms to light, leading to reduced system efficacy (tailing effect)^13^. Previous work has demonstrated reduced UV effectiveness due to elevated p-m association, even when turbidity is low^15^. For all mechanisms, electrostatic attraction and repulsion forces between microorganisms and particles plays a key role in the extent and stability of p-m associations^16^. Zeta Potential (ZP), the electrical potential between the slipping plain of a particle and the bulk water, has recently been attracting attention as a key indicator of p-m association^17,18^. One potential approach to disrupting p-m associations and enhancing UV inactivation of water with elevated particulate matter is the manipulation of ZP.

ZP can be effectively manipulated using three main methods: chemical additives, pH adjustment, and external electric fields. pH adjustment can influence the electrostatic interactions and stability of particles by modifying their surface charges. Introducing chemicals such as polymeric compounds can change the ZP by creating bridges between particles, neutralizing surface charge, or forming a three-dimension networks that trap microorganisms and particles. Applying an external electric field can alter the behavior and interactions of particles or microorganisms through polarization effects and electrical double-layer compression. These techniques provide powerful tools for controlling ZP and subsequently modulating particle behavior in various systems^19,20^. ZP measurement and control have been applied to enhance various water treatment processes such as coagulation-flocculation^21–26^ electrocoagulation^27^, adsorption^28^, membrane filtration^29^, and Dissolved Air Flotation (DAF)^30^ systems.

Previous studies have investigated the role of ZP or energy required to break particle association in water^31^. In a study by Liu et al. (2007), ZP showed a linear ascending relationship with the energy barrier that led to a log-linear decrease of aggregation rate. It is important to emphasize that this study exclusively focused on the positive range of ZP values, with high ZP indicating a significantly positive ZP. Mamane et al. (2006) investigated the impact of organism-organism and organism-clay aggregates on the degree of organism protection from incident light. It was concluded that the highest level of protection (up to 50%) was seen in organism-clay samples where aggregation was maximized with optimized flocculation^5^. The application of pre-treatment focused on ZP modification for improved UV disinfection remains largely unexplored despite the role of ZP and p-m association on UV dose-response^17,32^. While previous studies have monitored the levels of ZP in various systems, studies on the direct manipulation of ZP and the consequential effects have not been reported. Nevertheless, it is theoretically possible to eliminate or reduce p-m association by manipulating ZP.

The central hypothesis of this study is that controlling ZP can enhance the performance of UV treatment by reducing the association between particles and microorganisms. A UV-LED system was used to carry out inactivation experiments to investigate the impact of ZP control on UV response and p-m association. The biodosimmetry results were assessed using an established double exponential UV disinfection kinetic model. The effects of ZP manipulation on water characteristics, including particle size, mean count rate, pH, turbidity, and UV absorbance (UVA) are also identified. The raw intake water of a drinking water treatment plant that applies UV without pre-filtration was used to more accurately represent the realistic temporal/seasonal differences between natural water and pure water, that is often used in biodosimetry studies. Additionally, the study aims to explore the differences in the behaviour of viruses and bacteria in relation to ZP manipulation. This study bridges the gap between advanced UV treatment and challenges in small-scale water treatment by providing insight into the potential of ZP control as a viable approach for mitigating p-m association effects.

## Experimental

### Water sample Preparation and characterization

Water samples were collected from the headgates of a treatment plant located in Lavington, BC with design capacity of 160 million liters per day (ML/day) within the Greater Vernon area. This treatment plant does not have a filtration process prior to UV disinfection. To investigate the influence of ZP change in a water sample containing natural particulates, the sampling point was intentionally selected to be before all pre-treatment steps. Although dissolved components (e.g., dissolved organic and inorganic ions) in the water could impact the measurement of ZP, the water quality was kept constant in all experiments, and the samples were collected on a single day. Consistency were made sure by using water collected during a single sampling event. Although live microorganisms could reproduce over time, experiments were initiated promptly to minimize variations.

*Escherichia coli* (*E. coli*: American Type Culture Collection (ATCC) 15597) and bacteriophage MS2 (ATCC 15597-B1) were selected as surrogate organisms due to their common use as microbial indicators, effective representation of other pathogens, and compatibility with previous studies^33^. 5 mL of MS2 bacteriophage stock (at an initial concentration of 10^10^ PFU/mL) and 5 mL of *E. coli* stock (at an initial concentration of 10^10^ CFU/mL) were added to 495 mL of raw grab samples, resulting in an initial microorganism concentration of 10^8^ PFU/mL or 10^8^ CFU/mL for MS2 and *E. coli*, respectively. The samples were then stirred using a magnet stirrer at 50 rpm for 30 min to facilitate potential p-m associations.

To establish ZP control, 100 mL of the original water sample along with microorganisms were distributed in 200 mL beakers. To shift ZP toward positivity polyaluminum, chloride (PACl) coagulant used at the treatment facility (supplied by Brenntag Canada) was added to the samples at concentrations ranging from 0 to 30 mg/L. A 1 M sodium hydroxide solution was prepared in reverse osmosis water (NaOH pellets from Thermo Scientific Chemicals and used to shift the ZP towards negativity. In addition to the initial ZP of the samples (negative), three different ZP levels toward positivity (moderately negative, neutral and positive) were produced from each sampling set. Finally, the ZP was established at 5 data points for each sampling set, comprising viral surrogates in sample set 1 and bacterial surrogates in sample set 2. The samples were stirred for 30 min to ensure thorough mixing, followed by water quality characterization to assess the resulting changes. Then, 20 mL of the stirred samples were placed in 70 mm Petri dishes for UV dose-response experiments. It should be noted that the solutions with altered Zeta Potential were irradiated with UV without any prior sedimentation.

Water quality characteristics, including turbidity, ultraviolet transmittance (UVT), ultraviolet absorbance (UVA), pH, zeta potential, mean particle count rate, and average particle diameter of the samples, were recorded before and after the settling period. The turbidity measurement was from the stirred solution, taken from the middle of the sample in 200 mL beakers. Particle size distribution, mean count rate of particles and ZP were measured using a Zetasizer Nano ZS (Malvern Instruments, Ltd., Westborough, MA). UVT was measured using an Aqualog spectrometer (HORIBA Scientific, Piscataway, NJ). A VWR Turbidity Meter (Thermo Scientific, PA) was used to measure sample turbidities, and pH measurement was carried out using Thermo Scientific Orion 3-Star Benchtop pH Meter. The UV light intensity was measured using an ILT2400 Optical Light Meter to determine the UV intensity delivered at surface of water. The ILT2400 light meter was used to confirm surface irradiance but was not continuously monitored during irradiation. The device was calibrated per manufacturer standards at the time of use. All water quality measurements were taken in triplicate. Each experiment involved triplicate sampling from a gallon of water collected from the treatment plant. Each experiment involved triplicate sampling from a gallon of water collected from the treatment plant. For each test, three water samples were prepared, and their concentrations were measured to ensure consistency. The average value of these measurements was calculated as one replication. This procedure was consistently applied across all experiments.

### Surrogate microorganism enumeration

The concentration of microorganisms in water samples was enumerated before and after ZP change to capture the impact of additives on microorganisms and catch the potential detrimental effects of the adjustment. Microorganism enumeration was also conducted after the settling period (for p-m association quantification), and at each interval of UV exposure time (for disinfection efficacy). Propagation methods for both organisms followed the ATCC manuals specific to each strain. To enumerate the organisms, 20 µL of samples were collected and serially diluted to achieve countable concentrations, followed by the appropriate plate counting procedure steps depending on the surrogate microorganism. Concentrations ranging between 50 and 100 counts per plate were deemed appropriate for accurate enumeration. Conducting the study on two different microorganisms under the same experimental conditions was aimed to facilitate acquiring reliable information and was essential for understanding the dynamics and associations between microorganisms and particles in water, enabling optimized and fit-for-purpose design and management strategies^19^.

#### MS2 enumeration

The Double-Layer Technique was used to estimate the concentration of MS2^34^. The method involves creating a double agar layer system where the bottom layer consists of a nutrient agar medium (solidified Luria-Bertani broth (LB) − 1.5% agar (Miller formulation; Fisher Scientific)), and the top layer is made up of a soft agar medium (LB-0.6% agar, with 45–50 °C temperature range maintained for the soft agar) containing 50 µL of the serially diluted MS2 sample to be tested and the 50 µL of the host bacteria (*E. coli*). After incubation (18 h, 37 °C), observed plaques identified as clear circular zones were counted, multiplied by dilution factor (the ratio of the final volume of MS2 in the tested sample to the volume of the original solution used to dilute it) and divided by the sample volume in millilitre to determine the titter in plaque-forming-unit per (PFU/mL).

#### *E. coli* enumeration

The *E. coli* enumeration was carried out by the Spread Plate Method^34^. To enumerate *E. coli*, 50 µL of the serially diluted water sample, was spread evenly across the surface of the solidified LB with 1.5% agar (Miller formulation; Fisher Scientific) using a sterile spreader, ensuring uniform coverage of the agar surface. After spreading the sample, the agar plates are incubated at 37 °C for 18 h. *E. coli* colonies appear distinct, circular, and often slightly raised on the agar surface. These colonies were counted after the incubation period. Considering the dilution factor and the volume of the sample, the concentration of *E. coli* was calculated in colony-forming units per milliliter (CFU/mL).

### P-m association quantification

Pre-tests using reverse osmosis (RO) water and treatment plants water with natural particles were conducted to ensure the relevance of the p-m association observed in our study. A settling-based methodology was applied to enumerate the portion of microorganisms associated with particles. A settling time of 30 min was selected to differentiate the free-floating MS2 and *E. coli* from the attached organisms (after gently stirring for 2 h). It should be noted that the 30-minute settling time was solely intended for enumerating associated organisms and should not be considered part of the treatment train. The settling method was based on a previous study by Farrel et al.^18^ to maintain relevance to the existing body of knowledge in the field.

The settling method used in this study to quantify p-m associations has certain limitations, as there may be remaining microorganisms in the supernatant that are associated and protected. Additionally, free organisms may be carried by the vertical gravitational force of sweeping flocs and particles. Nevertheless, when applied correctly and consistently in all experiments, this method can provide a reliable and relative estimate of associated particles in the samples. The percent particle-associated microorganisms were calculated using Eq. (1). After the settling step, the concentration of microorganisms in the supernatant was enumerated as the free-floating microorganism. The concentration of all present microorganisms (free-floating and particle-associated) were calculated from the enumeration of the sample before settling time. The percentage of p-m association was determined based on the reduction in microbial concentration after ZP modification, reflecting the protective role of particles, regardless of the number of organisms associated.

1 $$\:\text{\%}\:association=\:=\left(\frac{{{C}_{o}-C}_{supernatant}}{{C}_{o}}\right)100$$

where C_o_ is the initial concentration of samples, and C_supernatant_ is the concentration of the supernatant of samples after the settling period.

### UV exposure and disinfection kinetic model

A UV-LED system (PearlLab BeamTM, AquiSense Technologies) was used to carry out inactivation experiments. After sample preparations, 20 mL of the stirred samples were placed in 70 mm Petri dishes for UV dose-response experiments. The UV light source was positioned at a distance of 9.4 cm from the water surface, and the irradiated path length (depth of water) was 0.6 cm. During experiments, the water was gently stirred to ensure uniform dose distribution. Inactivation curves (dose-response) were generated based on the average of the three replicates. The remaining samples were allowed to settle for 30 min to obtain the settled samples. After the settling period, 10 mL of the supernatant was gently extracted for water quality characterization and microorganism enumeration in the supernatant. Water samples containing MS2 and *E. coli* were irradiated at a wavelength of 255 nm with an average intensity of 478 µW/cm^2^. In all experiments, samples were exposed to four different doses from which 20 µL of the sample was taken at each dose level to conduct serial dilution and microorganism enumeration. All dose-response measurements were taken in triplicate for each sample replicate.

The reduction in the abundance of bacteriophage (N) due to UV exposure can be described by the double-exponential model (Eq. (2)) as follows^35^:

2 $$\:N=\left(1-\beta\:\right){N}_{\text{o}}{e}^{-{k}_{1}{UV}_{Dose}\:}+\beta\:{N}_{\text{o}}{e}^{-{k}_{2}{UV}_{Dose}\:}$$

where, N is the residual concentration of culturable organisms after UV exposure, N_o_ is the initial number of culturable organisms in PFU-CFU/mL, and UV_Dose_ is the delivered UV Dose in mJ/cm^2^. In addition, β indicates the fraction of UV-resistant organisms, and finally k_1_, and k_2_ represent first-order inactivation rate constants for UV-susceptible and UV-resistance fractions, respectively. The use of a double-exponential model allows for the representation of tailing effects, or concavity to the dose-response model, that are commonly observed from UV disinfection.

The actual UV dose delivered to the samples can be calculated by Eq. (3)^36^. This calculation were made following the Bolton and Linden (2003) protocol^37^.

3 $$\:UV\:Dose=Es\:Pf\:\left(1-R\right)\frac{L}{d+L}\frac{\left(1-{10}^{-UVA\:254\:d}\right)}{{UVA}_{254}\:d{ln}\left(10\right)}t$$

where, Es is the average UV intensity (mW/cm^2^), Pf is the unitless Petri factor, R is the reflectance at the air-water interface at 254 nm, L is the distance from the lamp centerline (cm), d is the depth of the suspension (cm), UVA 254 is the UV absorbance (UVA) at 254 nm (calculated by Eq. (3)), t is the exposure time (s). The optical light meter reading recorded an E_s value of 0.478 mW/cm^2^. The Pf factor was measured at 0.99, while the reflectance (R) was 0.025. The distance (d) was 0.52 cm, and the length (L) was 9.4 cm.

UVT and UVA can be related by a logarithmic function as Eq. (4).

4 $$\:UVA=\text{l}\text{o}\text{g}\:10\:(1/\text{U}\text{V}\text{T})$$

### Statistical analysis

Statistical significance of the results was investigated using Student’s t-test. It was performed in pairs for the double-exponential model’s β, P, k_1_, and k_2_ terms Eq. (2) at a 95% confidence level. The difference between two samples was deemed to be statistically significant if the calculated p value was ≤ 0.05. A two-way (with replication) ANOVA at a confidence level of 95% was used to determine the impact of ZP change and UV dose on the inactivation of surrogate organisms. Statistical analyses were conducted using Python, employing libraries such as Pandas, NumPy, Scikit-learn, and Statsmodels.

## Results and discussion

### Sample characterization

Water samples were collected from a treatment facility that utilizes a UV disinfection system to process unfiltered water, and the collected samples closely represent the raw water source. The general quality characteristics of the natural raw water, including pH, hardness, Dissolved Organic Carbon (DOC), alkalinity, UVA, turbidity, average particle size and ZP used for this study is provided in Table 1. This data has been acquired through in-house measurements as well as communication with the treatment plant.

**Table 1:** Water quality characteristics of the background Raw water.

Water quality characteristics	Unit	Value
pH	–	7.20
Conductivity	µS/cm	50.00
Hardness	mg/L as CaCO₃	23.40
DOC	mg/L	9.10
Alkalinity	mg/L as CaCO₃	21.90
UVA	–	0.04
Turbidity	NTU	1.30
Average particle size	µm	2.80
Zeta potential	mV	− 15.80

**Fig. 1:**
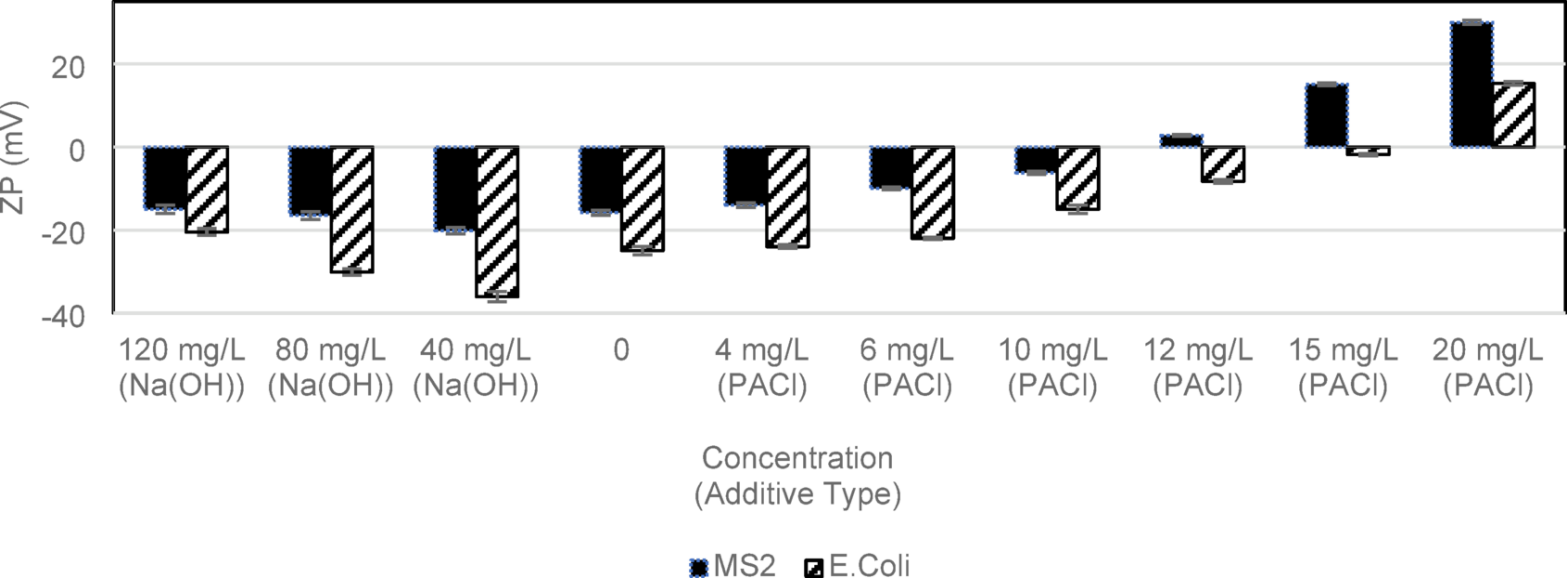
The impact of additive dosage on the ZP behaviour of raw water spiked with MS2 (solid fill) and E. coli (diagonal strips). Error bars represent one standard deviation over replicates (*n* = 3).

Polyaluminum chloride (PACl) coagulant and a 1 M sodium hydroxide basic solution were introduced separately to water samples. The purpose was to adjust the ZP towards positivity and negativity, respectively. Figure 1 illustrates the relationship between ZP and the doses of additives. The patterns observed in ZP are influenced by the initial water quality and the specific microorganisms present^38^. Nonetheless, a general and direct parabolic relationship between coagulant dose and ZP can be observed where it reaches its negative peak at 40 mg/L NaOH and positive peak at 20 mg/L of coagulant. Similar relationships with ZP were observed in a study by Wei et al. when PACl coagulant was added at dosages of 0 to 20 mg/L^39,40^. PACl, at the concentration used, did not cause a significant pH change. However, during ZP changes with pH alteration due to energy barrier shifts at particle surfaces. Initially, increased pH lowers ZP by reducing the energy barrier. However, once saturated, further pH increases can transition ZP from negative to positive^41^. Liu et al. link these changes to H⁺ and OH⁻ ion adsorption: H⁺ ions shift ZP positive, while OH⁻ ions decrease ZP. Multivalent ions in surface water, especially at higher pH, can neutralize surface charges and affect ZP^42^. In the MS2 samples, the pH reached a value of 11 at the highly negative ZP range (− 25 to − 40 mV), while the *E. coli* samples at the sameZPrange exhibited a pH of 8.6. For the rest of the ZP ranges, the samples maintained relatively stable pH values, with an average of 6.9 and a standard deviation of 0.27.

In this study, the primary aim of ZP control was to manipulate the dominant electrostatic forces between the particulates and control the potential associations. Extreme levels on opposite sides of the ZP axis were achieved in the laboratory. The typical dose applied at the treatment facility is 40–70 mg/L PACl for reduction of NOM by dissolved air flotation. Disregarding the objective of NOM reduction, for the objective of ZP control, a lower-than-usual (up to 20 mg/l) coagulant dose achieved up to + 15 mV of ZP without triggering significant formation of flocs. Observations from experiments (data is not presented) and supporting literature^39^ confirm this conclusion Five final ZP levels were selected for the experiments to cover a range of conditions and varying degrees of p-m association.

Due to slight variations in the obtained ZPs for both sample sets, the ranges are categorized as highly negative (ZP = − 26 to most negative achievable), negative (ZP = − 16 to − 25 mV), moderately negative (ZP =− 3 to − 15 mV), neutral (ZP = − 2 to + 2 mV), and positive (ZP = + 3 to the most positive achievable). The neutral category interval was chosen to have a variation of ± 2 mV, consistent with the observed standard deviation of 1.84 (*n* = 3) achieved when applying the same dosage to attain neutrality in samples.

**Fig. 2:**
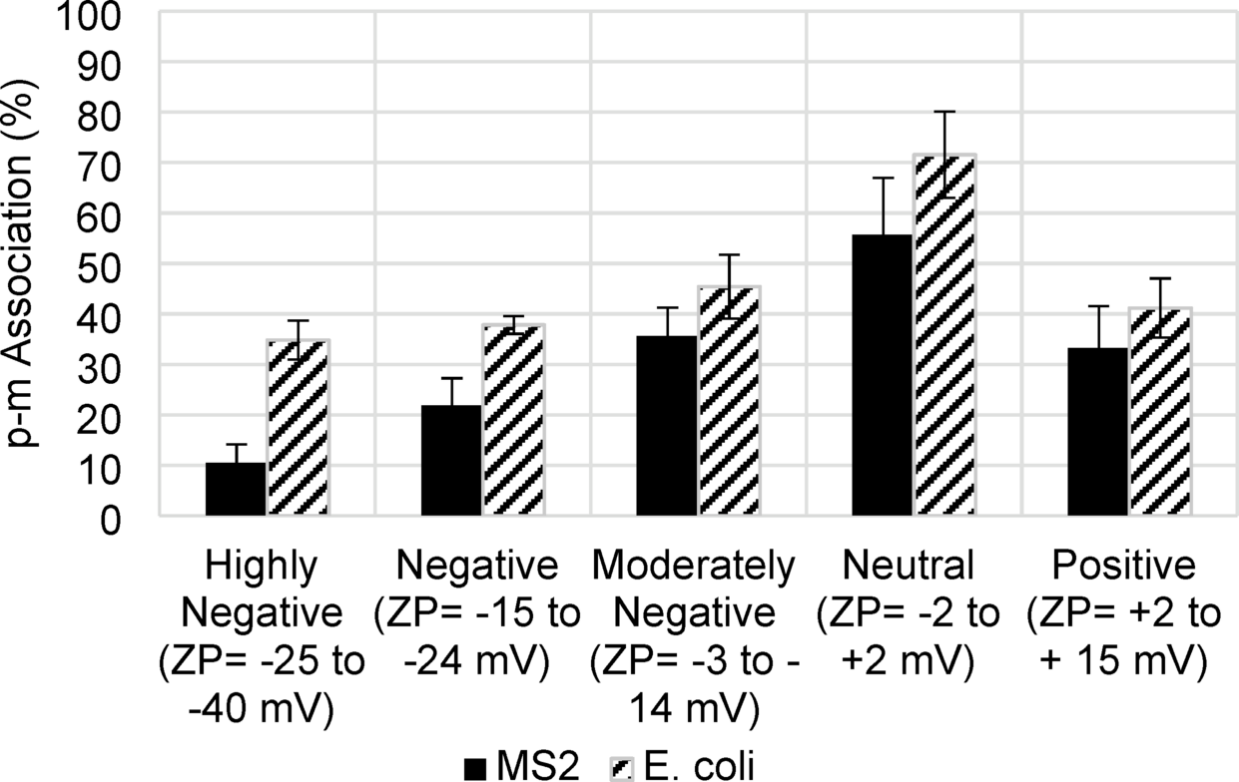
p-m association (%) level of MS2 (solid fill) and E. coli (diagonal strips) sample sets at different ZP levels measured by settling separation methods. Error bars are the standard deviation of three replications.

**Fig. 3:**
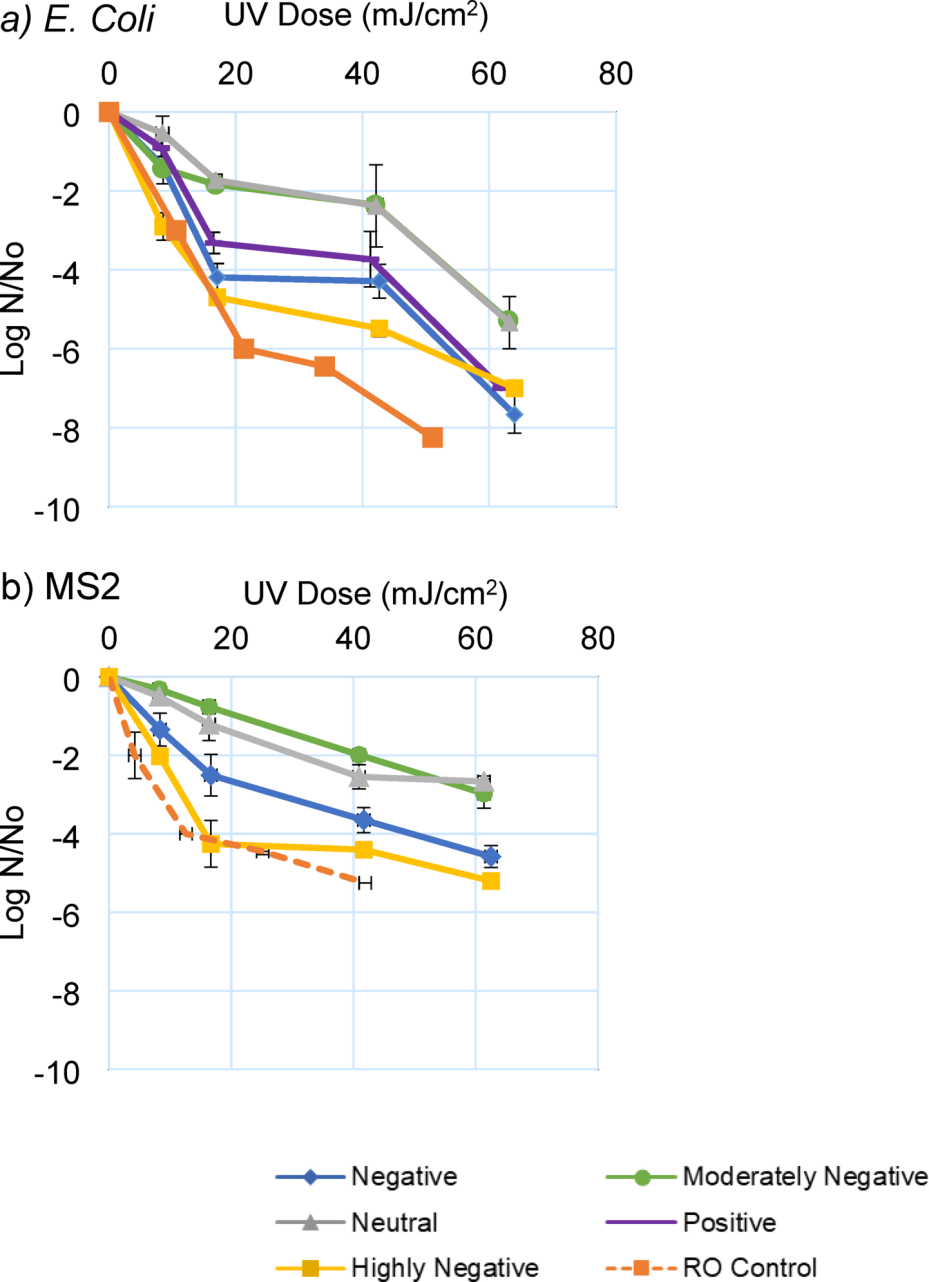
UV inactivation efficiency of (**a**) E. coli and (**b**) MS2 surrogate microorganisms. Control tests are representative of organism inactivation in RO water sample with no additive addition. Error bars represent one standard deviation (*n* = 3).

Different microorganisms resulted in varying ZP in the final solution (Table 2). Changes in the ZP parameter were accompanied by corresponding changes in other water quality indicators. The surface charge characteristics and the interactions between the microorganisms and the water matrix depend on various factors, such as the composition of the microorganism’s cell wall, the presence of functional groups on the cells^43^, the pH, and ionic strength of the water^44,45^. MS2 and *E. coli* were added separately to simulate microbes in source waters and compare system responses, maintaining a constant microorganism concentration across experiments. Figure 1 illustrates that MS2 exhibits a less negative ZP than *E. coli*. The MS2 bacteriophage is significantly smaller and has a protective coating that is distinct from the bacterium *E. coli*. When both are present at the same concentration (10^8^ CFU or PFU/mL), the difference in surface charge can contribute to the observed variations in ZP (Fig. 1). The introduction of MS2 led to the original raw water becoming 7.39 mV more negatively charged, while *E. coli* induced a greater change, causing it to become 16.59 mV more negative. Despite the significant differences in ZP caused by these organisms, the observed trends in p-m association (Fig. 2) and UV disinfection (Fig. 3) efficiency remained consistent, irrespective of the organism type. Therefore, the results can be applied to different situations; however, site-specific studies are necessary for precise adaptation.

**Table 2:** ZP values achieved at each sample in the relevant ZP range.

ZP range	MS2	E. coli
	ZP (mV)	ZP (mV)
Highly negative (zeta potential= − 25 to − 40 mV)	− 20.13	− 36.03
Negative (zeta potential= − 15 to − 24 mV)	− 15.8	− 24.93
Moderately negative (zeta potential= − 3 to − 14 mV)	− 6.21	− 8.25
Neutral (zeta potential= − 2 to + 2 mV)	2.77	− 1.81
Positive (zeta potential = + 2 to + 15 mV)	15.1	15.3

**Fig. 4:**
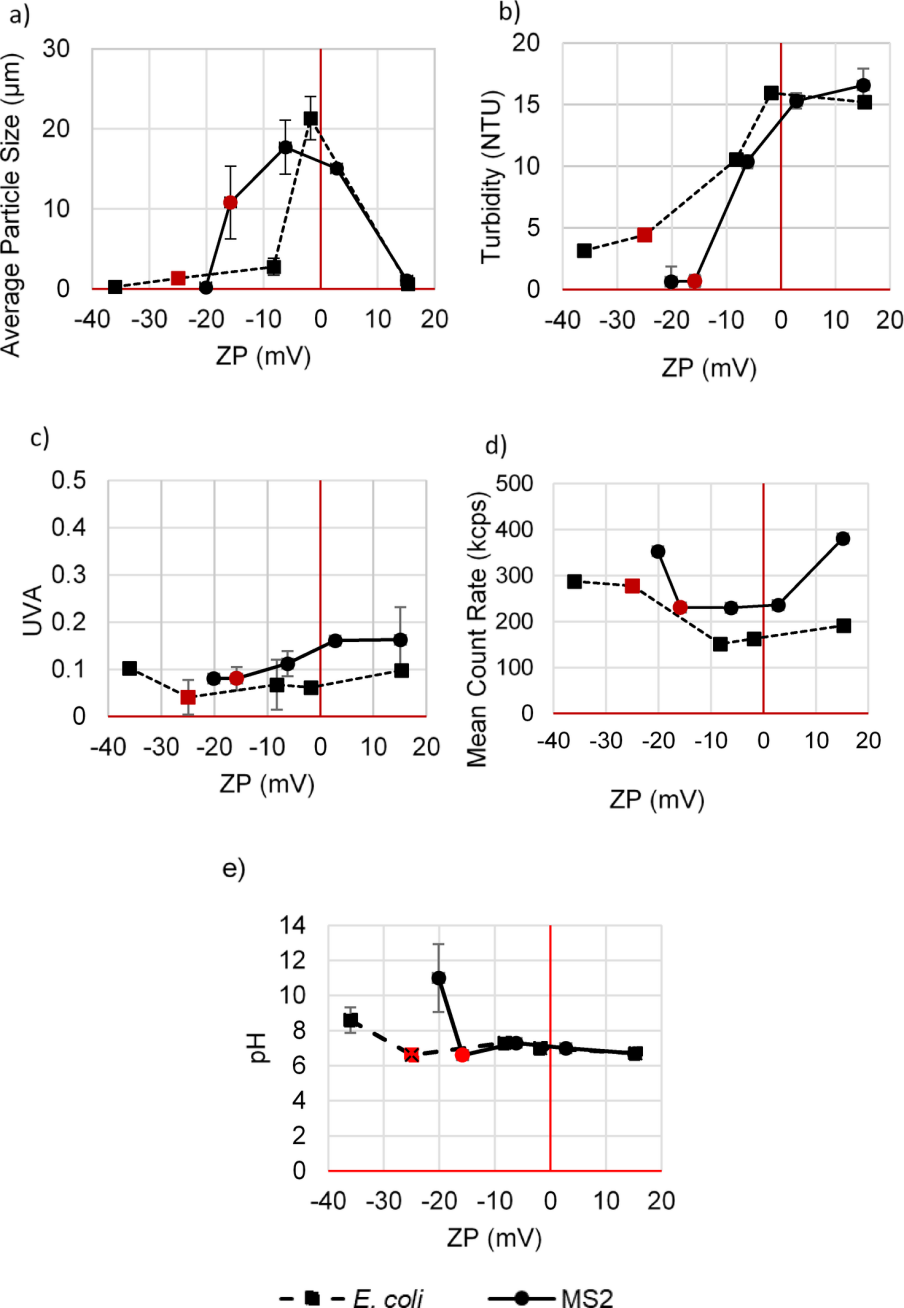
Water quality characteristics of samples at ranges of ZP in samples spiked with MS2 (Solid line) and E. coli. (Dashed line): (**a**) average particle size (µm), (**b**) turbidity (NTU), (**c**) UVA, (**d**) mean count rate (kcps), and (**e**) pH. Samples were made in triplicates and the error bar represents the standard deviation of replicates (*n* = 3). The red dots represent the control sample (sample with no ZP manipulation).

The water quality characteristics (ZP, UVA, Turbidity, mean count rate, pH and average particle size) of both control samples (before additive addition: red dots in Fig. 4) and samples after ZP control were monitored (after additive addition: black dots) are shown in Fig. 4. The results show distinct patterns in water quality changes as ZP was altered, with variations depending on the additive dose and the type of microorganism present. Seasonal variation in water quality could occur in real-world scenarios, and the significance of this variation can be substantial. The optimal additive amount to achieve the desired ZP is likely to be site-specific and dependent on dynamic source water conditions. However, the required dose is measurable via bench-scale tests and periodic adjustments would be possible based on seasonal changes.

The average particle size (Fig. 4a) increased when the ZP approached neutrality but decreased as the absolute value of ZP increased. Turbidity (Fig. 4b) increased with the increase in ZP up to neutral (up to 15 NTU) and levelled off at positive ZP. Increasing turbidity can be attributed to the imposed aggregation and weak floc formation by the added coagulant. The addition of coagulant initiated an immature coagulation and flocculation process by neutralizing charges and promoting particle aggregation. However, the system is under-dosed compared to optimal floc formation, resulting in weak and poorly formed flocs with lower structural integrity that would be more prone to breakage and dispersion^46^. It was observed that samples with MS2 at moderately negative ZP (− 3 to − 15 mV) resulted in larger particle sizes due to better neutralization (Fig. 4a). Bacteria typically range in size from 0.5 to 5 μm, whereas the MS2 bacteriophage is significantly smaller, typically reported as 25–30 nm^47^. Less coagulant was needed to neutralize MS2 than *E. coli*, as their initial ZP was less negative. In contrast, at the neutral ZP (~ 0 mV) the highest particle size was seen in *E. coli* samples with larger size regardless of the initial ZP. Despite being in the same ZP category, differences were observed between the aggregation levels of organisms based on changes in average particle sizes. A general trend from previous work suggests larger organisms have a higher likelihood of aggregation with larger size aggregates^48,49^ in neutral samples^49^. MS2 samples achieved neutrality more effectively than *E. coli*, as their initial ZP was less negative. This proximity to neutrality resulted in improved aggregation with particles. In contrast, at the neutral ZP (~ 0 mV) the highest particle size was seen in *E. coli* samples with larger size regardless of the initial ZP.

Determining whether particles aggregate with themselves or other substances is essential, especially in high-concentration experiments. Xing et al. found that aggregation and surface interactions occurred when MS2 viruses were exposed to colloids. While agglomeration was possible, its significance varied depending on factors such as particle shape and Derjaguin-Landau-Verwey-Overbeek (DLVO) interactions. The DLVO theory explains the stability of colloidal particles in suspension by considering the balance between attractive van der Waals forces and repulsive electrostatic forces arising from the electrical double layer surrounding the particles^31^. Their study also revealed that even in high MS2 concentrations (e.g., 10^9^ PFU/mL), most MS2 are strongly bound to colloids, impacting their behaviour during fate and transport^50^. In a study by Langlet et al., it was noted that MS2 aggregation started effectively at pH < 3.9^51,52^. While water samples in the present study were maintained at relatively higher pH levels (7–11), the aggregation under those conditions is assumed to be minimal. However, there is a pressing need to understand further colloidal interactions between water quality indicators and different organisms to improve the current water treatment processes.

As the ZP moved towards neutrality, in MS2 samples, UVA (Fig. 4c) slightly increased (+ 0.08 cm^− 1^) and then remained constant from the neutral to the positive side of the ZP range. However, for *E. coli*, the trend in UVA demonstrates a consistent gradual increase of + 0.05 cm^− 1^ from the control sample to the positive ZP. When coagulant (PACl) is added to the water, the formed positively charged aluminum hydroxide species can exhibit different optical properties compared to their uncharged counterparts^53^, causing these variations. When coagulant doses are sufficient for DOC removal, these species facilitate the destabilization and aggregation of particles and dissolved organic matter. Given the low doses of coagulant used, it was not expected that UVA would be increased significantly. Charge neutralization coagulation regimes are not overly effective for NOM removal, which is the major component contributing to UV absorbance^1^. It should be noted that despite the turbidity levels exceeding the recommended thresholds (recommended turbidity < 1.0 NTU) for UV reactors, UVA remains only slightly lower than the regulated threshold (recommended UVA < 0.125 equal to UVT > 75% or, but dependent on UV reactor manufacturer). The presence of adequate light transmittance is the key factor enabling disinfection to be achieved, even in highly turbid samples^54^.

The mean count rate (Fig. 4d) exhibited a decreasing trend towards neutrality in both sample sets, followed by an increase as the absolute value of ZP increased. The count rate represents the frequency at which particles or microorganisms were detected during the measurement in counts per second. The count rate for *E. coli* was lower compared to MS2. The water samples were spiked with high concentrations of microorganisms (10^8^ PFU and CFU/mL) to create controlled and reproducible conditions with enhanced sensitivity to high log inactivation. Therefore, samples were saturated with microorganisms that could induce high levels of organism aggregation and p-m association, especially in *E. coli*, where larger organism sizes led to lower counts per second. *E. coli* may have a lower count rate due to its larger size^55^, or its larger size may lead to aggregation^56^, which also results in a lower count rate.

One crucial aspect to consider in these experiments is comparing adjustments made to pH levels (in a more basic direction) and adjustments made using PACl. The addition of a basic solution to create samples with highly negative ZPs has shown limited influence on the monitored quality characteristics in this study. However, manipulation of ZP by basic solution significantly increases the pH levels. In contrast, quality characteristics changed when PACl was added to the water, but the pH remained relatively stable. Similar results on pH level of water were observed in the study by Wei et al. (2015) when PACl coagulant was added at dosages of 0 to 20 mg/L (Fig. 4e)^39^. The processes and interactions due to both methods (NaOH and coagulant addition) are interconnected. Changes in the surface properties of the particles due to pH change or coagulant addition can lead to changes in the size, count, and adsorption level of particles^57^. Changes in the pH can affect various chemical equilibria at the molecular level and result in calcium carbonate precipitation and changes to redox potential^58^. Therefore, when discussing the manipulation of ZP through manipulating pH, it is important to consider the overall effects high pH levels could have on the water quality. Furthermore, the UV filtering effect of dissolved organic matter (DOM) likely differs between these two scenarios. The addition of PACl can coagulate with dissolved organic matter (DOM), reducing its concentration in the solution and impacting the observed water quality characteristics. It was confirmed that MS2 remains viable at high pH, as demonstrated by both literature evidence^51,59^ and control pre-tests conducted in the lab. This study focused primarily on studying the direct effects of ZP adjustments on UV dose-response. The effects of DOM were considered through monitoring UVA (Fig. 4-c). UVT values (see Eq. (4)) were calculated and used as a key indicator for adjusting the delivered dose effectively and capturing the impact of water transmittance in the final UV disinfection achieved. Future work should explore the impact of DOM on ZP manipulation to provide a comprehensive understanding of these interactions.

**Fig. 5:**
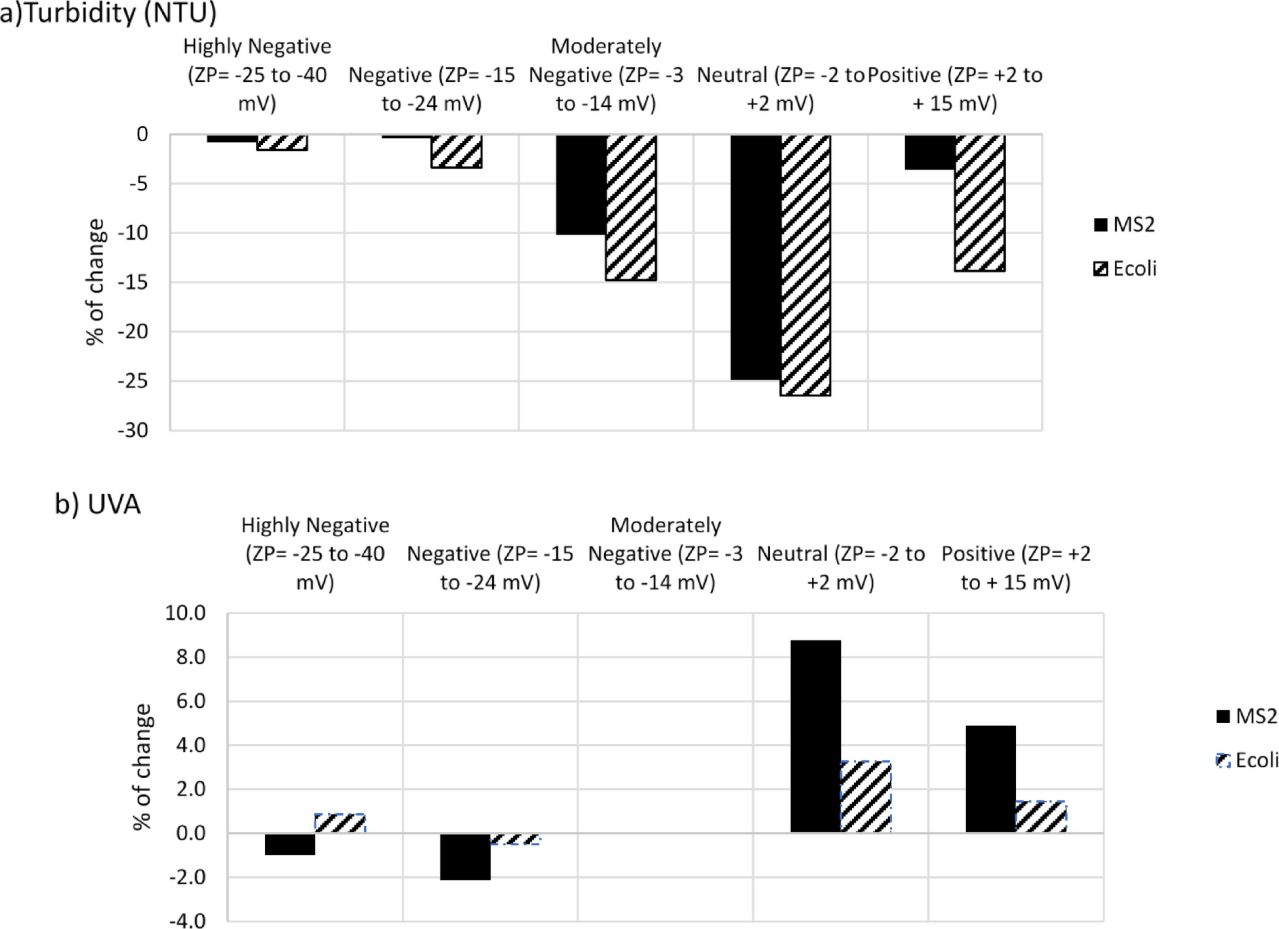
The difference between quality characteristics: (**a**) Turbidity and (**b**) UVA of water samples after settlement period. The negative values represent a drop and positive values represent an increase after settlement. The values represent the difference between the averages of three measurements where in all measurements the coefficients of variation were less than 5%.

To further support the weak interactions of particulates in positive ZP, the percentage of the difference between turbidity and UVA after the settling period is presented in Fig. 5. Samples were settled to allow the portion of associated microorganisms to settle due to gravity forces. The intention was to highlight the extent of change in the two most common UV water quality indicators (turbidity and UVA) resulting from the separation of associated organisms. Negative values indicate a decrease, while positive values indicate an increase following the settling process. The highest percentage of change in turbidity and UVA is observed in the neutral sample with the highest level of p-m association, consequently leading to their settling. The lowest absolute value of the percentage of change can be seen at the two far ends of the ZP axis where neutralization is minimal, and the water quality characteristics do not exhibit improvement (decrease in turbidity and in UVA compared to the original) after settling time. It is worth noting that settling has demonstrated no effect on the UVA of a sample when there was no manipulation of ZP (control sample). However, it does lead to a 10–15% decrease in turbidity. This implies that the organisms associated with particles in the control sample did not contribute to UVA. However, manipulating the ZP increased the absorbance and the loss in light transmission is considered a result of PACl addition.

### P-m association analysis

There are two physical approaches to separate and enumerate associated and non-associated organisms: filtration and settling. The settling method was selected as the primary approach for detecting p-m associations due to the ineffectiveness and inconsistency of the filtration technique. Specifically, after the addition of the coagulant, the filters became clogged, causing high variability and significant variance between replicates. This issue arose because coagulation altered the particle size (results can be seen in Fig. 4-a), making filtration unreliable.

A settling time of 30 min was selected to differentiate the free-floating MS2 and *E. coli* from the attached organisms after gently stirring for 2 h. The settling process yielded consistent and reproducible results for both organisms (Fig. 2). Notably, a higher level of p-m association (Eq. (1)) was observed in *E. coli* compared to MS2 in all levels of ZP. Both samples showed the highest level of microorganism association at neutral ZP. These findings are consistent with previous studies, further supporting the correlation between microorganism association and ZP levels^38^.

The consistently higher p-m association of *E. coli* compared to MS2, and the particularly strong association observed in basic solutions, can be attributed to differences in their isoelectric points (pI) and the resulting charge characteristics. MS2 has a reported pI of approximately 3.9^51^, indicating that it carries a positive charge in acidic environments and a negative charge at higher pH. *E. coli* has a pI range of 4.5–5.5^60^, rendering it less negatively charged at basic pH compared to MS2. Given that the particles in the sample are originally negatively charged, the less negative surface charge of *E. coli* reduces electrostatic repulsion, enabling stronger interactions with the particles. This difference in charge intensity may also promote secondary interactions, such as hydrogen bonding or Van der Waals forces, which are less dependent on direct electrostatic attraction. The increased p-m association for *E. coli* in basic solutions suggests that weakened electrostatic repulsion under these conditions allows alternative mechanisms—such as hydrophobic or steric interactions—to dominate. These mechanisms are likely less significant for MS2 due to its simpler structure and smaller size, which reduce its potential for such interactions.

### UV disinfection efficiency and inactivation rate constants

This study examined the efficiency of UV disinfection in terms of log reduction (logarithm of N/N_o_) for both MS2 and *E. coli* sample sets with varying ZP. All samples were exposed to UV for up to 150 s at a wavelength of 255 nm. UV dose is calculated as intensity multiplied by exposure time. Doses were corrected to estimate the actual UV dose delivered based on Eq. (3), which considers the effect of varying UVA between samples. The results of the UV experiments are presented in Fig. 3; Table 3 summarizes the parameters for each microorganism and ZP level, including β, K_1_, and K_2_. Generally, MS2 samples exhibited lower inactivation compared to *E. coli* across all ZP levels. Similar results were observed in previous studies showing higher overall bacterial susceptibility over viral surrogate susceptibility^61,62^. The lowest disinfection levels were observed in the samples with neutral and moderately negative ZP, which was consistent for both organisms. This observation can be attributed to the greater p-m association in the neutral and moderately negative samples (Fig. 2). Disinfection was greater for positive ZP samples than neutral/moderately negative ones, despite typical UV performance indicators suggesting water quality would be most challenging (turbidity at maximum, UVA at maximum for positive ZP samples; Fig. 4). Tailing effects were also observed in the RO control to some extent (proportion of UV-resistant organisms, β = 18%), even without particulates. The RO control refers to a test where only reverse osmosis (RO) water and microorganisms were used, with no particulates or ZP manipulation, to assess disinfection rates in the absence of particulate matter and to consider the level of aggregation that might occur under these conditions. Aggregation of organisms in the RO control sample can increase their chances of survival^63^. The consistent microbial concentrations in the initial samples suggest that variations in UV disinfection efficacy were primarily due to p-m associations, as aggregation was intentionally controlled for in our study design. As such, the results suggest that accounting for p-m association enhances our understanding of UV performance, and UVA/turbidity may be inadequate at capturing the reduction in UV performance due to p-m association. High spiking concentrations were necessary to ensure measurable microbial counts post-UV exposure, with control experiments confirming minimal aggregation or particle association.

**Table 3:** Values of the double exponential model parameters (n-3, R2 < 0.9).

	Parameters	ZP				
		Highly negative (ZP= -26 to -40 mV)	Negative (ZP= -16 to -25 mV)	Moderately negative (ZP= − 3 to − 15 mV)	Neutral (ZP= − 2 to + 2 mV)	Positive (ZP = + 2 to + 15 mV)
MS2	β	19.00	61.82	84.94	78.84	71.42
	k_1_	**−** 0.90	**−** 0.26	**−** 0.90	**−** 0.15	**−** 0.20
	k_2_	**−** 0.21	**−** 0.26	**−** 0.11	**−** 0.15	**−** 0.20
E. Coli	β	0	9.00	8.85	73.65	56.43
	k_1_	**−** 0.89	**−** 0.90	**−** 0.90	**−** 0.18	**−** 0.29
	k_2_	**−** 0.49	**−** 0.17	**−** 0.07	**−** 0.18	**−** 0.29

**Fig. 6:**
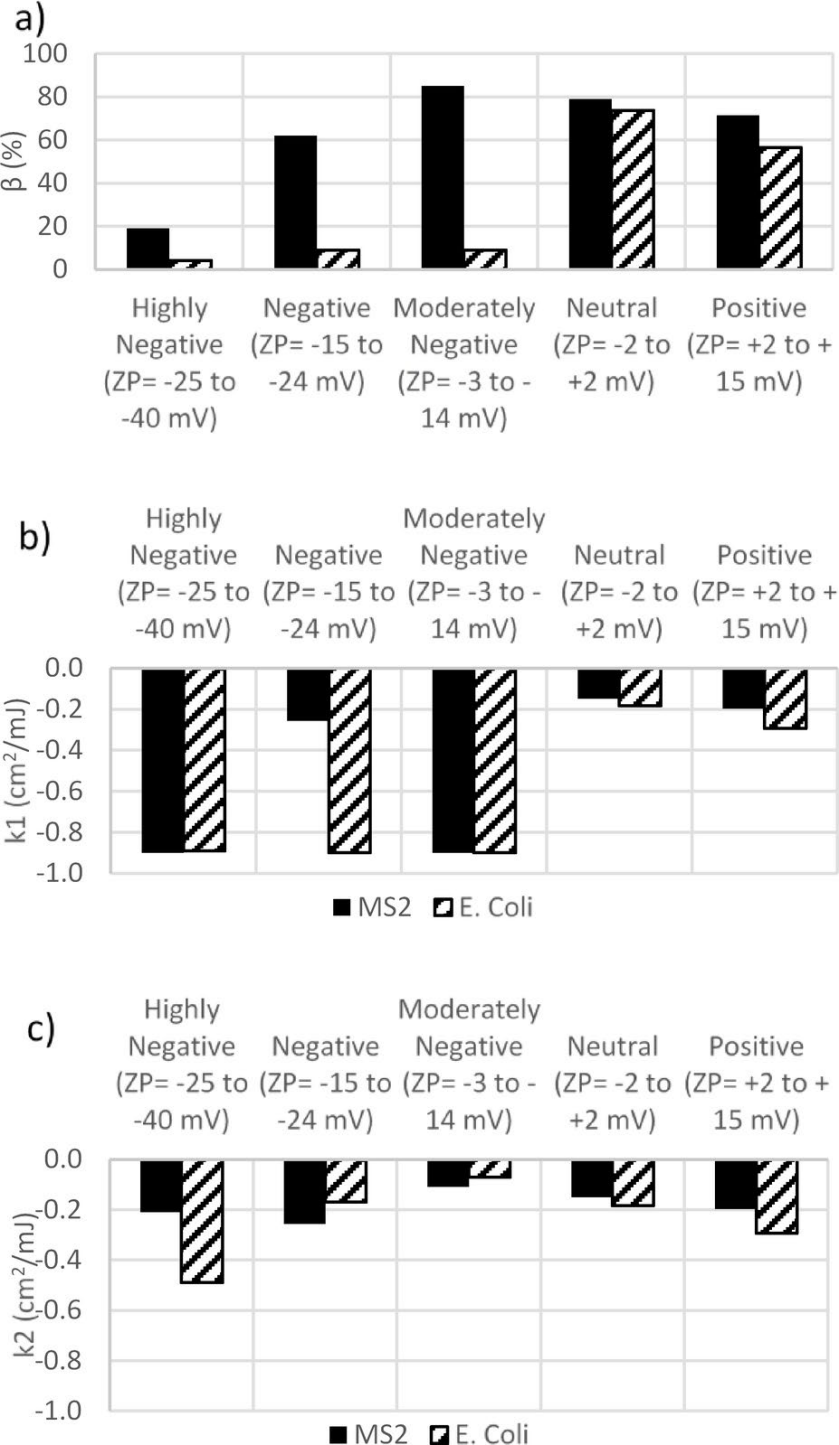
Double exponential model parameters over ZP control. (**a**) β is representative of the theoretical p-m association level in %, (**b**) k_1_ is the inactivation rate constant of easily disinfected free-floating organisms and (**c**) k_2_ the inactivation rate constant of the particle-associated organisms exhibiting resistance to UV. The R-squared values for all regression models were above 0.90.

The double exponential model for disinfection dose-response (Eq. (2)) was fit using experimental data to describe the disinfection process and differentiate between microorganisms contributing to tailing phenomena (all models fit with adjusted R^2^ = 0.85–0.99). Implementing this model allowed estimation of the inactivation rate constants associated with free-floating vs. particle-associated microorganisms and the percent of microorganisms contributing to tailing (β). The first parameter (k_1_) of the model represents the inactivation rate constant of easily disinfected free-floating organisms, while the second parameter (k_2_) of the model signifies the inactivation rate constant of the particle-associated organisms exhibiting resistance to UV. The parameters of the double exponential model are presented in Fig. 6. In MS2 samples, β exhibited a parabolic trend (Fig. 6-a), reaching its peak at moderately negative (β = 84.9% at ZP=-6.2 mV) and the lowest level in highly negative samples (β = 61.8% at ZP = -25.7 mV). For *E. coli* sample sets the lowest β was also seen in moderately negative (ZP= -24.9) sample at 8.85% and the highest at neutral ZP (β = 73.6% at ZP= -1.8 mV). It should be noted that the observed 4-log reduction in clean water at 18 mJ/cm^2^ appears to deviate from the typical 18 mJ/cm^2^ per log reduction for MS2 at 254 nm. This discrepancy may be attributed to several factors, including the extremely high initial concentration of MS2 (10^8^ PFU/mL) used in our experiment, which likely resulted in a higher log reduction at a given UV dose compared to natural water conditions.

The theoretical p-m association level (β) trend is consistent with the results of experimental measurements presented in Fig. 2. Overall, linear correlations between β and p-m association for each individual organism were moderate to high and positive (MS2 *R* = 0.81; *E. coli* *R* = 0.77). However, there are inconsistencies considering patterns between organisms, with the experimentally determined p-m association levels suggesting greater levels of p-m association in *E. coli* compared to MS2 and an opposite trend indicated by fitted β values. The inconsistency between theoretical and laboratory particle association levels might be due to the different p-m mechanisms these approaches can cover. The changes to light received by the organisms due to particles and other constituents can be influenced by various mechanisms, including even the protective effects that may arise from shadowing organisms without any actual attachment. However, the experimental approach for detecting microorganism associations is limited to instances where organisms have organic material deposition or have physically attached themselves to particles. As a result, the method effectively captures scenarios only where these microorganisms settle due to the influence of gravitational forces. This phenomenon is more pronounced in the case of MS2 organisms with smaller sizes compared to *E. coli* which influences their sedimentation behavior and interaction with particles. Smaller microorganisms like MS2 are less likely to settle due to gravitational forces alone and are more prone to remain suspended in the water column, potentially leading to greater exposure to light. Additional biological mechanisms, such as differences in surface properties or attachment behavior, could also play a role in this effect. While these factors were not the focus of this study, they warrant further investigation.

The inactivation rate constant for the portion of free-floating organisms is represented as k_1_. The lowest k_1_ level was seen in neutral samples regardless of the type of the organisms (Fig. 6b). In theory, p-m association should only impact k_2_. However, altering ZP and p-m association also affected k_1_ values. The impact on k1 could be due to the partial protection of organisms (even if they were not included in p-m measurements) or other changes in water quality that were not captured in UV dose adjustment. Within a broader context of real-world conditions, disinfection-resistant strains (whether associated with p-m or not) within the population can further contribute to discrepancies in inactivation rates. In the neutral samples, the p-m association was most pronounced; therefore, the lower rate of inactivation was achieved where the organism was protected. The high p-m association indicates strong protection for a significant portion of the organisms present. However, the smaller fraction left free remained vulnerable to inactivation but with a lower rate. Similar findings were observed in a study by Mounaouer et al., where the effect of particles on the inactivation rate diminishes with increasing initial free-floating concentration^64^.

*E. coli* consistently showed higher disinfection rates than MS2, which is consistent with previous results. The rate for inactivation of microorganisms associated with particles (k_2_) was different depending on the organism type (Fig. 6c). k_2_ was greatest in highly negative *E. coli* and negative MS2 samples. The results suggest that the highest level of inactivation for associated organisms that are more negative (*E. coli*) occurred in more negative ZP. In the samples with less initially negative organisms (MS2), higher disinfection was achieved in negative ZP. Therefore, the optimum ZP to achieve the lowest p-m association and higher inactivation rate constants are organism-dependent. Complex systems often entail underlying dynamics and interactions that the model may not comprehensively capture. Additionally, the model relies on assumptions and simplifications to maintain tractability, but these simplifications may not fully encapsulate the intricacies of real-world scenarios.

Although positively charged *E. coli* samples show a stronger p-m association, the k_1_ and k_2_ values are significantly higher than those for MS2. This discrepancy could be attributed to the higher effectiveness of UV disinfection for *E. coli* in positive cases of ZP than in moderately negative ones. This observation suggests that attachment or enmeshment of organisms may be less pronounced or that the organisms are not as effectively protected under positive *E. coli* conditions.

## Conclusions

The effects of ZP control on water quality, p-m association, and UV disinfection were explored. The results indicated that neutral ZP led to the highest level of p-m association, irrespective of the type of microorganism, and was correlated with decreased UV performance. Moreover, the kinetic studies conducted in this research supported the conclusion that water samples with a highly negative ZP exhibited reduced tailing phenomena and higher UV efficiency, contributing to improved disinfection outcomes. Results suggest that ZP strongly impacts the effectiveness of UV disinfection and has poor correlations with measures used to assess UV system performance, including UVA/UVT and turbidity. Manipulation of ZP to greater negativity increased UV disinfection performance for all organisms studied.

Several factors and limitations should be considered when interpreting the results and conclusions of this study. Variations in organism susceptibility to UV damage, concentrations of test organisms, and variability in plaque formation due to differences in experimental materials could result in inactivation rates not consistent with previous studies. Additionally, dose calculations for the petri dish represent calculated conditions based on the experimental set up that are an estimation of actual dose received. Furthermore, the control of ZP using varying chemical additions could have caused changes in the system not reflected in water quality tests. Despite these factors, the trends observed within this study remain internally consistent, as the same methods and conditions were applied across all test cases.

The presented approach was investigated to identify pre-treatment methodologies that could be employed by small systems using UV as the primary disinfectant. While other pre-treatment approaches, such as filtration, may provide greater benefits, they are not implementable in many scenarios. Filtration systems demand careful consideration regarding capital costs due to equipment procurement and potential infrastructure costs. Furthermore, ongoing operational aspects such as regular backwashing, filter media replacement, and monitoring contribute to the overall cost considerations associated with filtration systems^65^. Conversely, chemical dosing methods for ZP manipulation may potentially offer advantages in terms of cost, implementation feasibility and space requirements, mainly when applied in existing reservoirs or reactors with available mixing^66^. Moreover, chemical dosing systems generally require less space, presenting an advantage in installations with limited available footprint compared to filtration^67^. However, there are potential drawbacks associated with consistent chemical dosing. These include considerations such as the generation of chemical residuals, chemical handling risks, downstream environmental impact, and the possibility of corrosion in distribution systems. These aspects highlight the necessity for careful evaluation and mitigation strategies when employing chemical dosing methods for water treatment^68^.

The study demonstrated that manipulating ZP towards positivity with a coagulant negatively impacted water quality, leading to high turbidity and aggregate formation, which in turn reduced UV disinfection efficiency. Conversely, shifting ZP to extreme negativity via pH adjustment did not compromise water quality and actually improved UV performance, as shown by reduced tailing phenomena and enhanced disinfection outcomes. Results also suggest that high turbidity levels with low p-m association still resulted in efficient UV disinfection, emphasizing the critical role of p-m association in enhancing UV treatment beyond conventional factors like turbidity and UVA/UVT.

Implementing ZP control through pH adjustment offers a minimally intrusive alternative to advanced filtration techniques, particularly where filtration is impractical or economically impossible. Addressing the challenges posed by ZP manipulation involves exploring neutralization methods to mitigate potential downstream impacts such as increased infrastructure corrosion, scale formation, and altered microbial growth. Future research should focus on developing strategies to manage these adverse effects and integrating ZP monitoring within UV reactors. This approach, which requires straightforward monitoring and spatial considerations, could enhance water treatment processes by optimizing system performance and efficiency without the complexity of filtration.

## Data Availability

The datasets used and/or analysed during the current study available from the corresponding author on reasonable request.
